# Case report: Long-term survival of a pancreatic cancer patient immunized with an SVN-2B peptide vaccine

**DOI:** 10.1007/s00262-018-2217-x

**Published:** 2018-08-01

**Authors:** Hiroaki Shima, Goro Kutomi, Fukino Satomi, Masafumi Imamura, Yasutoshi Kimura, Toru Mizuguchi, Kazue Watanabe, Akari Takahashi, Aiko Murai, Tomohide Tsukahara, Takayuki Kanaseki, Yoshihiko Hirohashi, Yuji Iwayama, Tetsuhiro Tsuruma, Hidekazu Kameshima, Noriyuki Sato, Toshihiko Torigoe, Ichiro Takemasa

**Affiliations:** 1 Department of Surgery, Surgical Oncology and Science, Sapporo Medical University, S1, W16, Chuo-ku, Sapporo, 060-8543 Japan; 2 Department of Pathology, Sapporo Medical University, Sapporo, Japan; 3 Medical and Biological Laboratories CO., LTD., Sapporo, Japan; 4 Department of Surgery, Takikawa Municipal Hospital, Takikawa, Japan; 5 Department of Surgery, JR Sapporo Hospital, Sapporo, Japan; 6 Odori Breast Thyroid Gland Clinic, Sapporo, Japan; 7 Sapporo Dohto Hospital, Sapporo, Japan

**Keywords:** Pancreatic neoplasm, Vaccination, Cancer survivor, Immunology, Antigen

## Abstract

A 62-year-old woman who underwent surgery to treat pancreatic cancer provided written, informed consent to undergo adjuvant therapy with gemcitabine, tegafur, and uracil. However, this was stopped after only 14 days due to Grade 4 neutropenia. She was then started on vaccine therapy with Survivin 2B peptide (SVN-2B) including IFA and INF-α. Although metastatic lung tumors were identified and resected at 82 months after surgery, the patient has remained free of new or relapsed disease for 12 years thereafter. Tetramer and ELISPOT assays revealed the continuous circulation of SVN-2B-restricted cytotoxic T-lymphocytes (CTLs) in her peripheral blood, and CTL clones had specific activity for SVN-2B at 12 years after surgery. The adverse effects of the peptide vaccination were tolerable and comprised low-grade headache, nausea, and fatigue. A prognosis beyond 10 years in the face of pancreatic cancer with distant metastasis is extremely rare. This experience might indicate the value of cancer vaccination therapy.

## Introduction

Immune reactions against cancer cells depend on an acquired immune system that antigen-dependently recognizes target cells. Thus, peptide vaccination therapy is a rational approach to the enhancement of immune reactions against antigens expressed by cancer cells. An early review article described that peptide vaccination therapy elicits a response rate of only 2.6% [1]. However, various epitopes have since been derived from solid tumors and clinical studies of peptide vaccination therapy have generated promising results [2].

With the aim of promoting clinical applications for cancer peptide vaccination therapy, the HLA*24:02-restricted peptide, Survivin 2B 80-88 (SVN-2B), has been administered to patients with colon, breast, lung, oral cavity, urinary bladder, and pancreatic cancers in clinical trials since 2003 [3]. We investigated the effects of SVN-2B on pancreatic cancer between 2005 and 2010 (UMIN000000905) [4] and experienced a patient who was remarkable for being the only one of six similarly treated patients to achieve over 12 years of survival. Here, we describe the clinical course and distinctive immunological response of this patient.

## Case report

**Fig. 1:**
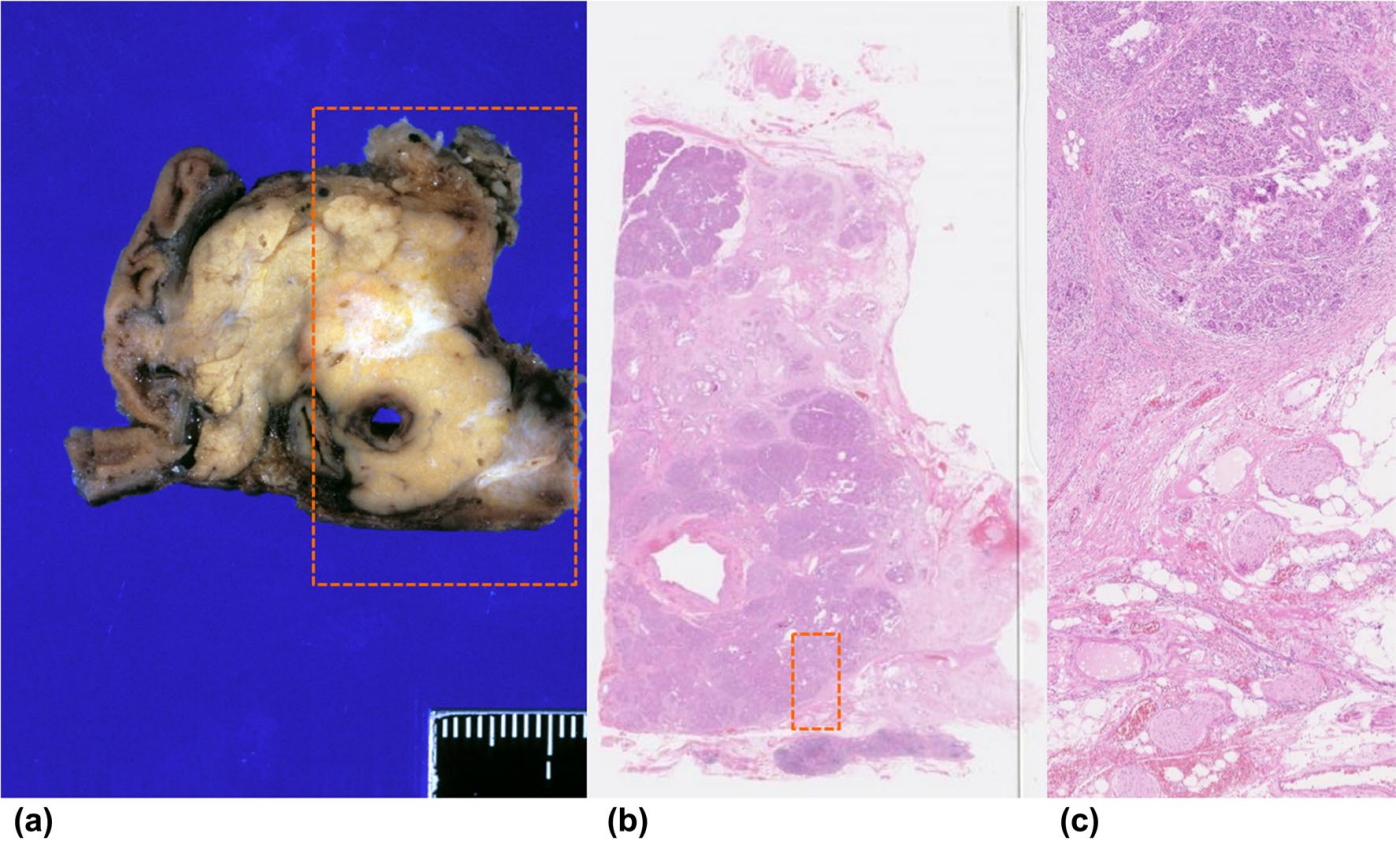
Pathological findings of pylorus-preserving pancreatoduodenectomy. **a** Left panel: formalin-fixed specimen of pancreatic head resected by PPPD. **b** Central panel shows main tumor (×40; H&E stain). **c** Right panel (dotted box) shows invasion of extra-pancreatic nerve (×100; H&E stain)

**Fig. 2:**
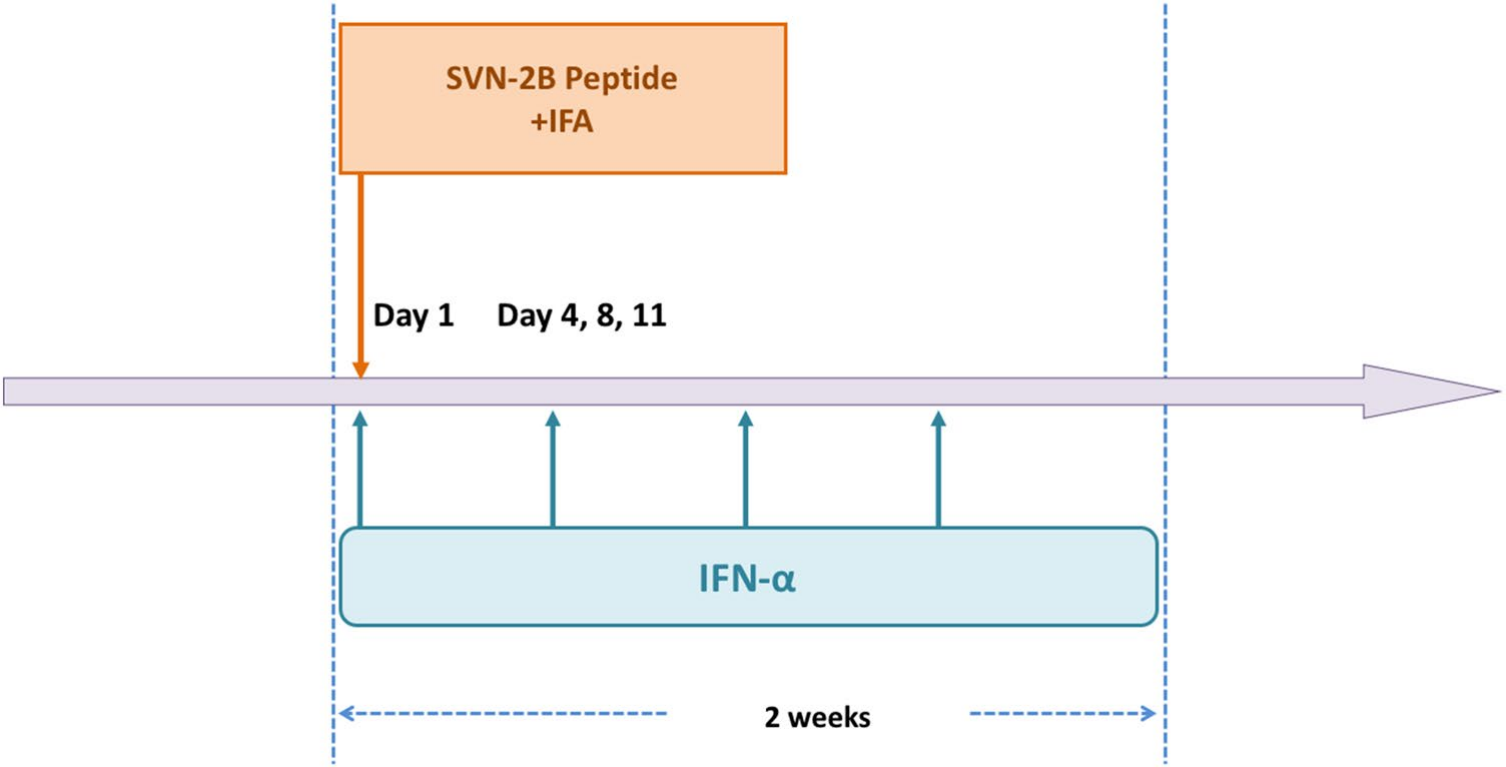
Vaccination protocol. SVN-2B vaccine and IFA were subcutaneously injected once every 2 weeks. Human IFN-α was simultaneously injected near IFA injection site on days 1, 4, 8, and 11. All three agents were repeatedly administered

**Fig. 3:**
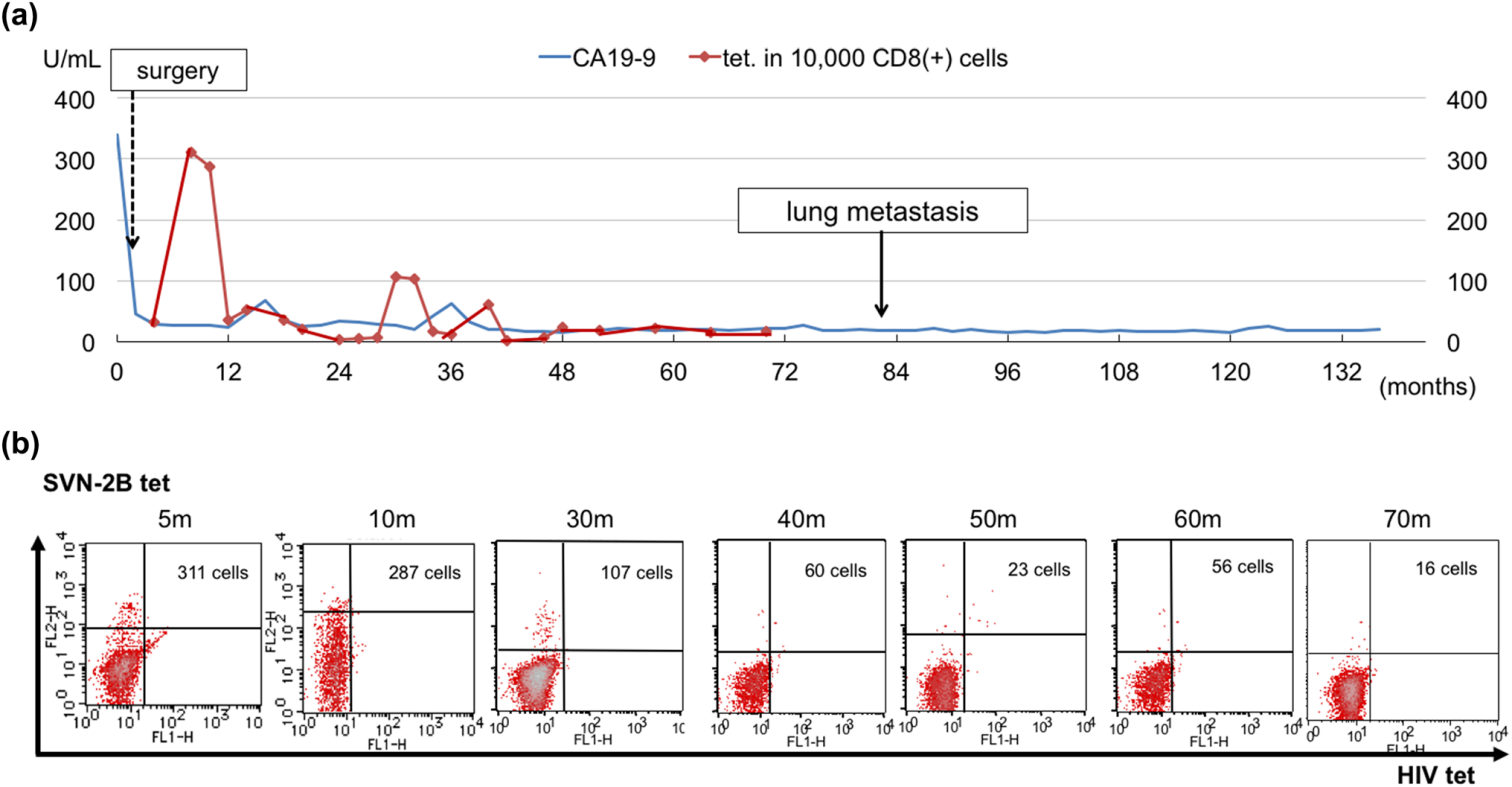
Longitudinal values for serum CA19-9 and tetramer-positive SVN-2B-specific CTLs. **a** Plasma CA19-9 did not increase and tetramer-positive cells continued to circulate for 12 years. **b** SVN-2B-specific CTLs were detected throughout the clinical course

A 62-year-old woman diagnosed with pancreatic cancer T3N0M0 Stage IIA [5] underwent pylorus-preserving pancreatoduodenectomy (PPPD) + D2. The pathological findings revealed invasive ductal carcinoma of the head of the pancreas, nodular and well-moderately differentiated type with a 3.8 cm diameter, with direct cancer cell invasion of the duodenal mucosa and extra-pancreatic nerve plexuses, as well as lymph node metastasis (#13b involving five nodes). The dissected peripancreatic tissue margin was positive for pathological stage III pT3N2M0 cancer [5] (Fig. 1a–c). The patient provided written, informed consent to undergo adjuvant treatment using gemcitabine, tegafur, and uracil (a 5-fluorouracil prodrug). However, this therapy was stopped after only 14 days as the patient developed Grade 4 neutropenia. Three months after surgery, the patient was enrolled in a clinical study of SVN-2B peptide vaccination. The vaccine at 1 mg/mL was mixed with the incomplete Freund’s adjuvant, Montanide ISA 51 (Seppic, Paris, France), emulsified, and then immediately injected subcutaneously once every 2 weeks. Human IFN-α at a dose of 3,000,000 IU (Dainippon-Sumitomo Pharmaceutical, Osaka, Japan) was also injected subcutaneously near the SVN-2B peptide injection site on days 1, 4, 8, and 11 (Fig. 2) [4, 6]. Serum CA19-9 levels did not increase over a period of 12 years postoperatively (Fig. 3a).

**Fig. 4:**
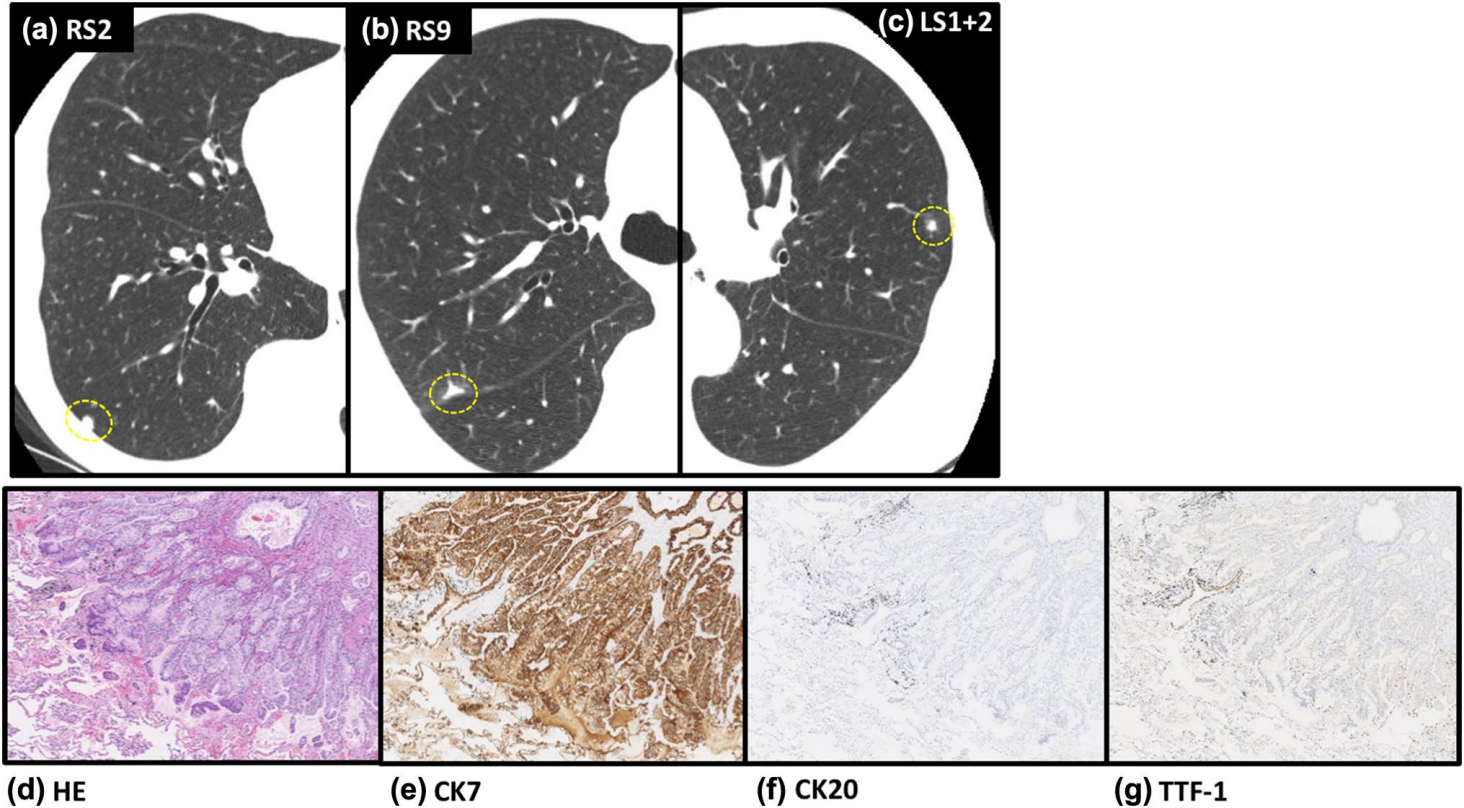
Details of metastatic lung nodules. **a**–**c** Three small nodules, in segments 2 and 9 of the right lung (RS2, RS9) and segment 1 + 2 of the left lung (LS1 + 2), detected in routine follow-up CT at 82 months after surgery. **d**–**g** All were pathologically well-differentiated adenocarcinoma and immunohistochemically positive for CK7 and negative for TTF-1- and CK20

A follow-up CT at 82 months after the surgery detected three small lesions (Fig. 4a–c) that were removed by thoracoscopic excisional biopsy. All of them were pathologically well-differentiated adenocarcinoma and immunohistochemically positive for CK7 and negative for TTF-1 and CK20, indicating that they were pancreatic tumor metastases (Fig. 4d–g). Twelve years after the primary operation, periodic computed tomography (CT) and positron emission tomography did not uncover any new local recurrences or metastases. The clinical course did not include severe adverse events, although low-grade adverse events included transient headache, nausea, fatigue, and a persistent red flare with induration at the injection site (Grade 1).

## Methods

### Tetramer assays and establishment of CTL clones

Flow cytometry proceeded using a FACSCalibur with CellQuest software (Becton Dickinson Biosciences, San Jose, CA, USA), FITC-labeled HLA-A*24:02-human immunodeficiency virus (HIV) peptide tetramer, PE-labeled HLA-A*24:02-survivin-2B peptide tetramer (MBL Co. Ltd., Nagoya, Japan), and PC5-labeled anti-CD8 antibody (clone T8; MBL) as described [4, 7]. Dead cells were eliminated as being 7-AAD positive.

We established CTL clones that were specific to SVN-2B peptide using SVN-2B tetramer as described [8]. In brief, PBMCs were stained with PC5-labeled anti-CD8 antibody and PE-labeled SVN-2B tetramer, then analyzed using a FACSAriaII flow cytometer (BD). CD8-positive and tetramer-positive cells were single-cell sorted into 96-well round bottom plates and cultured with 1 × 10^5^ irradiated (100 Gy) PBMC feeder cells in AIM-V medium (ThermoFisher Scientific, Waltham, MA, USA) supplemented with 10% human AB serum, 100 U of IL-2, and 1 µg/mL of PHA (Wako Pure Chemical Industries, Osaka, Japan). Thereafter, AIM-V supplemented with 10% HS and 100 U of IL-2 was added to the wells every 3–7 days. Cell growth was examined by tetramer analysis to determine SVN-2B specificity.

### Enzyme-linked ImmunoSpot (ELISPOT) and cytotoxicity assays

The specificity of SVN-2B-specific CTL clones was examined using ELISPOT assays as described [4, 7]. C1R cells (C1R-A24) transfected with HLA-A24 were pulsed with SVN-2B peptide (10 fg/mL to 10 µg/mL) and incubated with CTL clones, then IFN-γ-specific spots were visualized. The negative controls comprised C1R-A24 cells pulsed or not with HIV peptide.

The lytic activity of CTL clones was determined by CFSE-based cytotoxicity assays using Cytotoxicity Detection Kits (MBL) [9–12]. The negative controls comprised C1R-A24 cells pulsed or not with HIV peptide and K562 leukemia cells that are HLA-class 1-negative.

## Results

### Tetramer assays

**Fig. 5:**
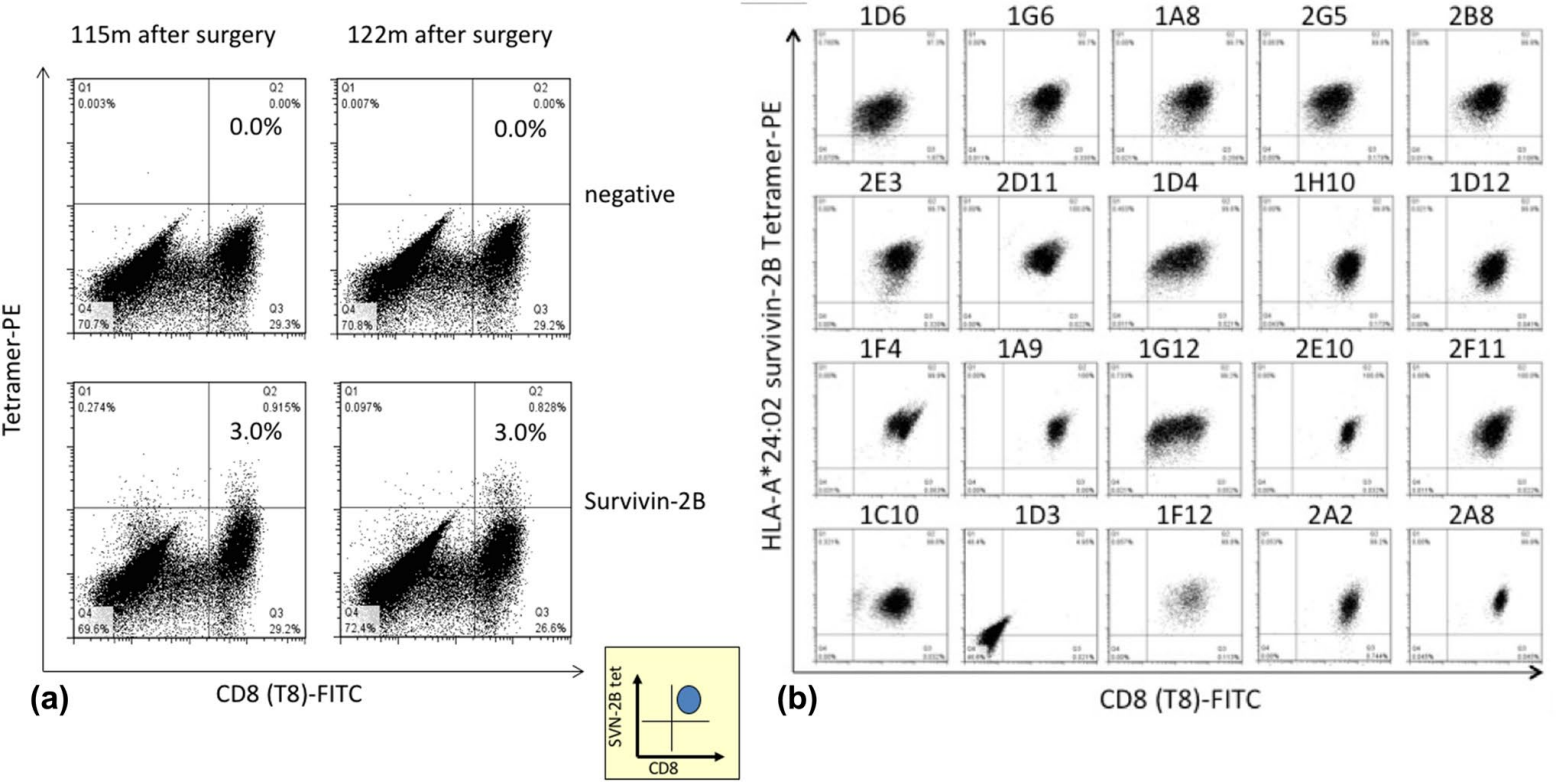
SVN-2B-specific CTL clones at 10 years after primary surgery. **a** Tetramer assay shows 3% of CTLs remain in circulation at 10 years after primary surgery. **b** CTL clones are positive for CD8 and HLA-A*24:02 SVN-2B tetramer in 19 out of 20 wells

SVN-2B-specific CTLs were detected throughout the clinical course between 5 and 122 months after the primary surgery (Figs. 3b, 5a), and tetramer-positive rates ranging from 0.16 to 3.11% were sustained at 3% even at 120 months thereafter (Fig. 3b).

### ELISPOT assays

**Fig. 6:**
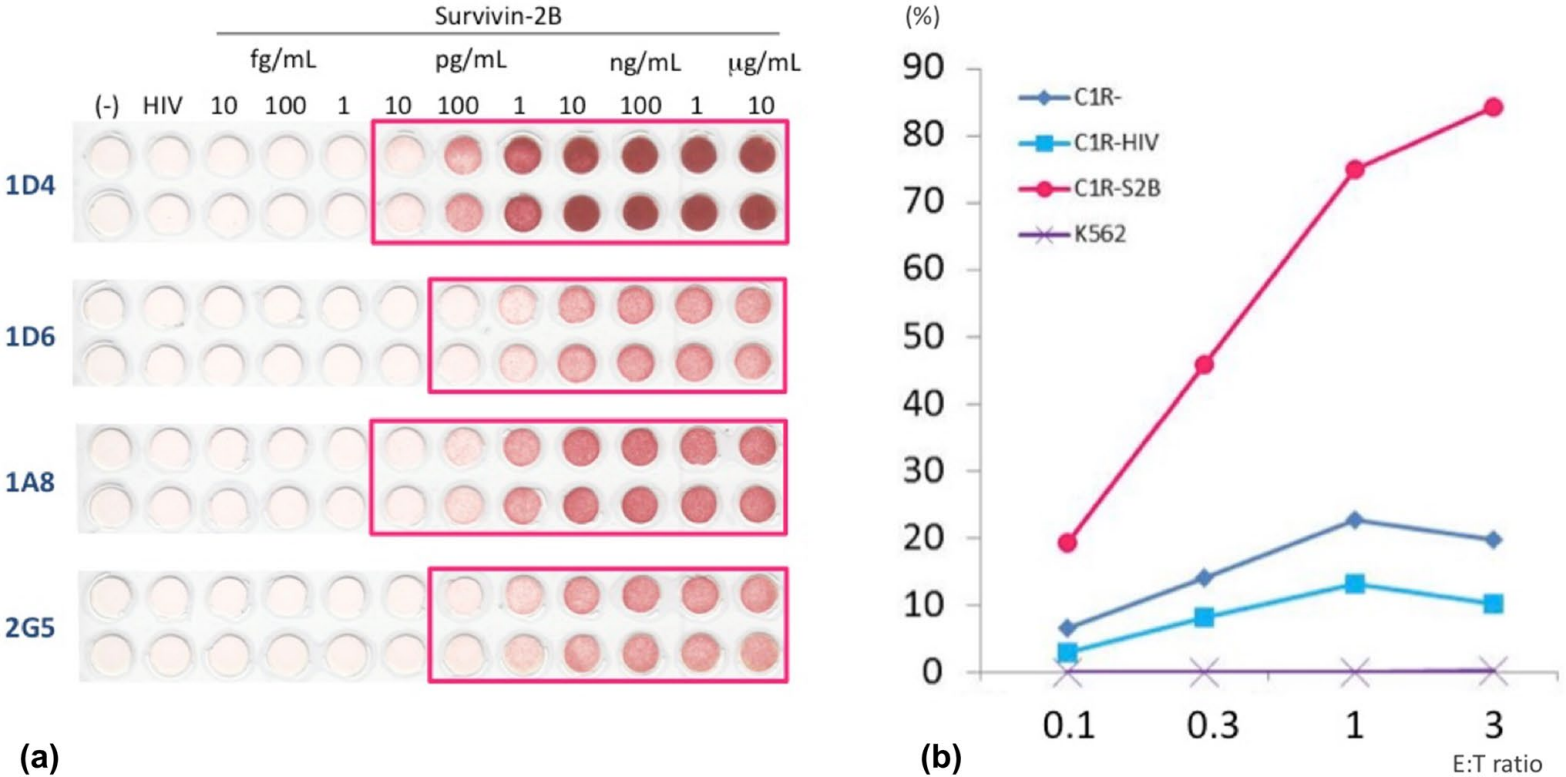
Specificity of SVN-2B-specific CTL clones. **a** ELISPOT results show that CTL clones dose-dependently respond to C1R-A24 cells at pg levels of SVN-2B. **b** CTL clones specifically indicate HLA-A24-restricted cytotoxicity against S2B (SVN-2B) peptide, and are unresponsive to K562 leukemia cells

SVN-2B-specific CTLs cloned from PBMC from the patient 10 years after the primary surgery were positive for both CD8 and HLA-A*24:02 SVN-2B tetramer in 19 of 20 wells (Fig. 5b) and dose-dependently responded to C1R-A24 SVN-2B in ELISPOT assays. These clones notably responded to C1R-A24 cells at the SVN-2B pg level (Fig. 6a).

### Cytotoxic assays

The CTL clones (1D4) specifically indicated HLA-A24-restricted cytotoxicity against SVN-2B peptide expression, and were unresponsive to K562 cells that did not express HLA-A24 (Fig. 6b).

## Discussion

This patient has survived for 12 years, despite having fatal, advanced pancreatic cancer. Few reports have described a prognosis beyond 10 years in the face of a pancreatic cancer diagnosis with distant metastasis. This particular patient could not tolerate adjuvant chemotherapy due to extremely adverse effects, and was therefore treated only with SVN-2B peptide vaccination for 12 years. Favorable survival rates in patients with isolated pulmonary metastases after pancreatic cancer surgery have been reported [13]. The median overall survival (OS) with isolated pulmonary metastasis in that study was 25.5 months (95% CI 19.1–31.8), suggesting that isolated pulmonary metastasis after surgery might be a favorable prognostic predictor. However, such patients rarely survive beyond 3 years, and none of the patients described in that report survived beyond 5 years [13]. The patient described herein developed pulmonary metastatic lesions in both lungs (Fig. 4a–c), indicating that these were not isolated pulmonary metastasis as described previously, yet she remains alive at 12 years after the primary surgery.

The present patient was registered in a phase 1 study described in our previous report [4], along with five other patients who also became eligible for treatment with SVN-2B. Tumor shrinkage and immunological effects were confirmed in all of them [4]. However, the median OS for these patients was only 5 (range 4–18) months, which was clearly not favorable. One explanation for this might be the metastatic involvement of more than one organ (lung, liver, peritoneum, and local) in these patients. Only the patient described herein had remarkably better OS among all patients in the phase 1 clinical trial. Liver metastasis has recently been associated with shortened progression-free survival and a reduced response and to anti-PD1 antibody treatment for melanoma and non-small cell lung cancer [14]. The infiltration of CTL was notably lower in patients with, than without metastasis to the liver and other lesions. The immunological mechanisms remain elusive, but the liver is associated with immune tolerance via several suggested mechanisms [15]. The better OS and the CTL response to SVN-2B peptide vaccination in the present patient might be associated with the absence of liver metastasis. The absence of liver metastasis among patients with advanced pancreatic cancers might be a good indication for peptide vaccination therapy in the future.

The phase III CapRI trial compared the outcomes of postoperative cisplatin, IFN α-2b, and 5-FU plus external radiation with those of 5-FU alone among patients with resected pancreatic adenocarcinoma [17]. Adjuvant chemoradioimmunotherapy notably did not improve the survival rate. The regimen in the CapRI study included 17 doses (3 doses/week) of 3 × 10^6^ units of IFN α-2b administered subcutaneously as adjuvant therapy and was completed about 1 month after primary surgery [16–18]. The median OS was 26.5 months in the experimental group with chemoradioimmunotherapy and 28.5 months in the control group with FU monotherapy, which represented the longest survival ever reported for patients in a randomized setting [16].

Immunotherapy also enhanced the quality of life and prolonged metastatic cancer-free survival in the present patient, whose background included a favorable metastatic site. SVN-2B-specific CTLs circulated in the peripheral blood of this patient for over 10 years, suggesting the induction of SVN-2B-specific memory T cells in vivo, namely a type I IFN-enhanced CTL response according to our previous study [4]. Thus, type I IFN might function not only in the induction of a CTL response, but also in the memorization of a cellular immune response. The longer period of peptide vaccination compared with adjuvant therapy might have ultimately contributed to the > 10-year survival of the present patient, distinguishing her from the other patients in the CapRI trial [16, 17].

The adjuvants administered to the present patient for immunotherapy were IFA and IFN-α. Adjuvants are considered necessary to induce a memory response, but most adjuvants have not received approval [19]. Adjuvants are ligands for receptors of the innate immune response system, and type I IFN is a major effector cytokine of adjuvants [20]. The course of this patient suggests that type I IFN might be sufficient to induce and maintain a memory response. The adverse effects of peptide vaccination were tolerable and comprised low-grade headache, nausea, and fatigue. These facts suggest that peptide vaccination with type I IFN might serve as a rational treatment for patients with postsurgical pancreatic cancer.

In summary, this report describes a unique patient with pancreatic cancer accompanied by distant metastasis who has been treated with peptide vaccination for 12 years. She remains free of cancer metastasis and recurrence, and has a good quality of life during the period.

## Abbreviations

CA 19-9: Carbohydrate antigen 19-9
CK: Cytokeratin
CTL: Cytotoxic T lymphocyte
ELISPOT: Enzyme-linked immunospot
FITC: Fluorescein isothiocyanate
HIV: Human immunodeficiency virus
HLA: Human leukocyte antigen
IFA: Incomplete Freund’s adjuvant
INF-α: Interferon alpha
PE: Phycoerythrin
SVN-2B: Survivin 2B 80–88
TTF-1: Thyroid transcription factor 1

## References

[1] Rosenberg SA, Yang JC, Restifo NP (2004). Cancer immunotherapy: moving beyond current vaccines. Nat Med.

[2] Pol J, Bloy N, Buqué A (2015). Trial watch: peptide-based anticancer vaccines. Oncoimmunology.

[3] Tsuruma T, Hata F, Torigoe T (2004). Phase I clinical study of anti-apoptosis protein, survivin-derived peptide vaccine therapy for patients with advanced or recurrent colorectal cancer. J Transl Med.

[4] Kameshima H, Tsuruma T, Kutomi G (2013). Immunotherapeutic benefit of α-interferon (IFNα) in survivin2B-derived peptide vaccination for advanced pancreatic cancer patients. Cancer Sci.

[5] Japan Pancreatic Society (2017). Classification of pancreatic carcinoma.

[6] Tanaka T, Kitamura H, Inoue R (2013). Potential survival benefit of anti-apoptosis protein: survivin-derived peptide vaccine with and without interferon alpha therapy for patients with advanced or recurrent urothelial cancer–results from phase I clinical trials. Clin Dev Immunol.

[7] Kameshima H, Tsuruma T, Torigoe T (2011). Immunogenic enhancement and clinical effect by type-I interferon of anti-apoptotic protein, survivin-derived peptide vaccine, in advanced colorectal cancer patients. Cancer Sci.

[8] Inoda S, Hirohashi Y, Torigoe T (2009). Cep55/c10orf3, a tumor antigen derived from a centrosome residing protein in breast carcinoma. J Immunother.

[9] Hirohashi Y, Torigoe T, Maeda A (2002). An HLA-A24-restricted cytotoxic T lymphocyte epitope of a tumor-associated protein, survivin. Clin Cancer Res.

[10] Sato T, Sato N, Takahashi S (1986). Specific cytotoxicity of a long-term cultured T-cell clone on human autologous mammary cancer cells. Cancer Res.

[11] Aubry JP, Blaecke A, Lecoanet-Henchoz S (1999). Annexin V used for measuring apoptosis in the early events of cellular cytotoxicity. Cytometry.

[12] Watanabe K, Suzuki S, Kamei M (2008). CD137-guided isolation and expansion of antigen-specific CD8 cells for potential use in adoptive immunotherapy. Int J Hematol.

[13] Kruger S, Haas M, Burger PJ (2016). Isolated pulmonary metastases define a favorable subgroup in metastatic pancreatic cancer. Pancreatology.

[14] Tumeh PC, Hellmann MD, Hamid O (2017). Liver metastasis and treatment outcome with anti-PD-1 monoclonal antibody in patients with melanoma and NSCLC. Cancer Immunol Res.

[15] Jenne CN, Kubes P (2013). Immune surveillance by the liver. Nat Immunol.

[16] Schmidt J, Abel U, Debus J (2012). Open-label, multicenter, randomized phase III trial of adjuvant chemoradiation plus interferon Alfa-2b versus fluorouracil and folinic acid for patients with resected pancreatic adenocarcinoma. J Clin Oncol.

[17] Märten A, Schmidt J, Ose J (2009). A randomized multicentre phase II trial comparing adjuvant therapy in patients with interferon alpha-2b and 5-FU alone or in combination with either external radiation treatment and cisplatin (CapRI) or radiation alone regarding event-free survival—CapRI-2. BMC Cancer.

[18] Knaebel HP, Märten A, Schmidt J (2005). Phase III trial of postoperative cisplatin, interferon alpha-2b, and 5-FU combined with external radiation treatment versus 5-FU alone for patients with resected pancreatic adenocarcinoma—CapRI: study protocol [ISRCTN62866759]. BMC Cancer.

[19] Khong H, Overwijk WW (2016). Adjuvants for peptide-based cancer vaccines. J Immunother Cancer.

[20] Seya T, Shime H, Takeda Y (2015). Adjuvant for vaccine immunotherapy of cancer–focusing on Toll-like receptor 2 and 3 agonists for safely enhancing antitumor immunity. Cancer Sci.

